# Tiotropium bromide as adjunct therapy in children with asthma: a clinical experience

**DOI:** 10.1186/s13223-021-00632-4

**Published:** 2021-12-12

**Authors:** Zainab Ridha, Marc-Antoine Bédard, Anna Smyrnova, Olivier Drouin, Aniela Pruteanu, Sandrine Essouri, Francine M. Ducharme

**Affiliations:** 1 Faculty of Medicine, Université Laval, 1050, Avenue de la Médecine, Quebec, QC G1V 0A6 Canada; 2 Clinical Research and Knowledge Transfer Unit On Childhood Asthma, Research Centre, Sainte-Justine University Health Centre, Montreal, QC Canada; 3 Department of Pediatrics, Centre Hospitalier Universitaire Sainte-Justine, Montreal, QC Canada; 4 Department of Pediatrics, Faculty of Medicine, Université de Montréal, Sainte-Justine Hospital, Montreal, QC Canada; 5 Department of Social and Preventive Medicine, Public Health School, University of Montreal, Montreal, QC Canada

**Keywords:** Asthma, Children, Tiotropium bromide, Therapeutic trial, Pediatric care, Case series

## Abstract

**Background:**

The Global Initiative for Asthma has only recently added tiotropium bromide as adjunct controller therapy in severe asthma (Step 4 or 5) in adults (2015) and children (2019). Although not yet approved for pediatric use by Health Canada, it has been occasionally offered by asthma specialists as a therapeutic trial in children with troublesome asthma or treatment for adverse effects. The objective of this study was to describe the indications and real-life clinical experience in initiating tiotropium in children with asthma.

**Methods:**

We designed a retrospective mixed-method case series study of children aged 1–17 years who initiated tiotropium in our tertiary-care centre between 2013 and 2020. Clinical information was extracted from electronic medical records and tiotropium dispensing, from drug claims. Parents/children and physicians independently completed a questionnaire about treatment goals, perceived efficacy, safety, satisfaction, and lessons learned.

**Results:**

The 34 (11 females; 23 males) children had a median (range) age of 9.1 (1.4–17.8) years. Children were primarily on Step 4 (85%) or 5 (6%) prior to tiotropium initiation, yet most (84%) did not increase their treatment step after tiotropium initiation. The physicians’ treatment goals were to improve asthma control, alleviate adverse effects of current therapy, and/or improve lung function. The most improved symptoms were coughing/moist cough, difficulty breathing, whistling breath, and bronchial secretions/mucus. Although most parents and physicians reported a significant benefit with tiotropium bromide, physicians particularly remarked, as their “lesson learned’, on the improvement in chronic symptoms in asthmatic children, particularly those with prominent moist cough and in lung function, in those with seemingly none (or incompletely) reversible obstruction as well as the ability to decrease the ICS and/or LABA dose to lessen adverse effects. A few physicians raised caution on the risk of lower adherence with an additional inhaler.

**Conclusion:**

In children with severe asthma on Step 4 or 5, tiotropium bromide was primarily used as substitute, rather than additional, adjunct therapy to improve asthma control, alleviate adverse effects, and/or to improve lung function. The latter two indications, combined with its perceived effectiveness in children with prominent moist cough, also suggest additional indications of tiotropium to be formally explored.

## Background

Tiotropium bromide (Spiriva®) is a long-acting muscarinic antagonist (LAMA) interfering with muscarinic M3 post-synaptic receptors; its use in asthma in adults has been associated with bronchodilation and decreased bronchial secretions, although it may also inhibit airway inflammation and remodeling [1, 2]. The comparable safety profile of tiotropium to placebo has been repeatedly demonstrated in children and adolescents [3, 4]. Since 2019, the international Global Initiative for Asthma (GINA) guidelines recommend considering tiotropium bromide as an add-on therapy to inhaled steroids (ICS) and long-acting beta_2_-agonist (LABA) as Step 4 (as an “other” controller option) or Step 5 (as “preferred” controller option, before considering biologics) therapy in children aged 6 years and older [5]. However, evidence supporting its efficacy in children less than 12 years old, particularly those less than 6 years, remains limited to a few trials [6–9]. While Canada and Japan have not yet authorized its use in children and adolescents [10, 11], tiotropium bromide has received regulatory approvals in children of 6 years and over in several countries, including the European Union (2015) by the European Medicines Agency [12], United States (2017) by the Food and Drug Administration [13], and Australia by the Therapeutic Goods Administration (2019) [14]; with a handful of countries authorizing its use in children as young as 1 year old (e.g., Chile, Peru). With only one trial [7], tiotropium uses in preschoolers is not currently mentioned in any guidelines [15–17], leaving scarce tested adjunct controller options for children with uncontrolled asthma in this young age group. In real-life practice, other indications may arise where tiotropium could be considered by asthma specialists, particularly in children with troublesome asthma and/or treatment adverse effects. Sharing this experience could generate new hypotheses regarding subgroups of patients or indications to support new research avenues.

The primary objective of this study was to describe, from a multi-faceted perspective, the indication and real-life clinical experience of initiating tiotropium bromide in pediatric patients treated in a Canadian tertiary-care pediatric asthma centre, from the perspective of physicians and families.

## Methods

We designed a mixed-method retrospective case series of children who initiated tiotropium bromide as a therapeutic trial at *Sainte-Justine University Hospital Centre* (SJUHC), a tertiary-care centre with a specialized asthma clinic. The Institutional Review Ethics Board of the SJUHC approved the study. In the context of the COVID-19 pandemic, electronic parent consent was obtained through the online platform *Lime Survey*. Parents who had previously provided written consent to enroll in the *Pediatric Asthma Database and Biobank* (PADB) of the SJUHC were approached for informed consent to this study; children 8 years and over provided assent. As all physicians who prescribed tiotropium during the study period were co-investigators, no consent was required for physicians participating in this study.

Children were eligible if they: (i) were aged 1 to 17 years of age, (ii) spoke French and/or English and had parents who spoke French and/or English, (iii) had a diagnosis of asthma confirmed by a medical specialist according to Canadian standards of practice [18, 19], (iv) received a prescription of tiotropium documented in the medical file, (v) had confirmed pharmacy dispensation of tiotropium confirmed by the Data Registry for Prescribed Medications (reMed) and (vi) had at least one follow-up asthma clinic visit three or more months after treatment initiation. Eligible patients with other chronic lung diseases were excluded.

The *Pediatric Asthma Database and Biobank* (PADB) was used to describe patients’ baseline (age, sex) and treatment (date of initiation, name, format, dose of medication and co-intervention, and treatment step both on arrival and discharge) characteristics. Briefly, the PADB, described in another study [20], is a structured electronic medical record with systematic documentation of sociodemographic characteristics; asthma diagnosis, phenotype, severity and control; pulmonary function tests results, when applicable; co-morbidities and environmental asthma triggers, as well as maintenance and/or rescue therapy reported on arrival to, and prescribed on discharge from, the clinic. The Data Registry for Prescribed Medications (reMed) provided data on all prescriptions served by any of Quebec’s pharmacies, claimed by privately or publicly insured patients was used to document pharmacy dispensing of tiotropium [21].

We designed a 14-item structured questionnaire to assess the children/parents’ and the treating physicians' perspectives regarding the indication and comfort level for tiotropium initiation, perceived efficacy and safety, reasons for, and outcome of, treatment cessation, perceived compliance with concurrent asthma medications, and satisfaction. The comfort level, as well as the overall assessment of efficacy, safety, adverse effects, and satisfaction, were assessed on a 7-point Likert scale, from − 3 (“significant deterioration” or “not comfortable at all”) to + 3 (“significant improvement” or “very comfortable”), with 0 meaning “neutral”. Children’/parents’ and physicians’ perceptions were collected independently, and children 8 years and over were encouraged to help complete the questionnaire with their parents. Participants could add comments to any of the survey’s questions. The children/parents’ and the treating physicians' questionnaires were almost identical, except for an additional open-ended question about lessons learned from the therapeutic trial asked only of physicians. Following the back-translation approach [22] and pretesting in parents and physicians, the electronic questionnaire was available in French and English.

Consenting parents and children over eight years of age were invited to complete the online electronic survey together. Alternatively, the questionnaire could be administered by telephone, in which participants were asked for permission to record the conversation to maximize accuracy. Participants received a 5$ compensation. The link to the questionnaire was sent to physicians by email, and they were encouraged to access the patient’s structured electronic medical chart to improve accuracy.

### Statistics

Patients’ characteristics, as well quantitative results, and responses from questionnaires, were reported with simple statistics: numbers and proportions for categorical variables and medians (IQR)/means (SD) for continuous variables. Data were analyzed using SPSS v.20 (IBM Inc.).

## Results

Between August 2013 and August 2020, 34 children with asthma were prescribed tiotropium; all were eligible to participate and had confirmation of drug dispensation by pharmacy records. Physicians completed the questionnaire for all 34 children. With 3 parents (9%) declining participation and 4 (12%) consenting without completing the questionnaire, 27 parental questionnaires were completed, 7 of which included the child’s input. The questionnaire was completed less than a year after tiotropium initiation in 6 (22%), between 1 to 2 years in 14 (52%) and more than 2 years in 7 (26%) patients; 18 parents (67%) reported that the delay did not affect their ability to answer the questions. Similarly, with access to medical records, physicians reported no interference of the delay on the perceived accuracy of their responses for all children.

**Fig. 1:**
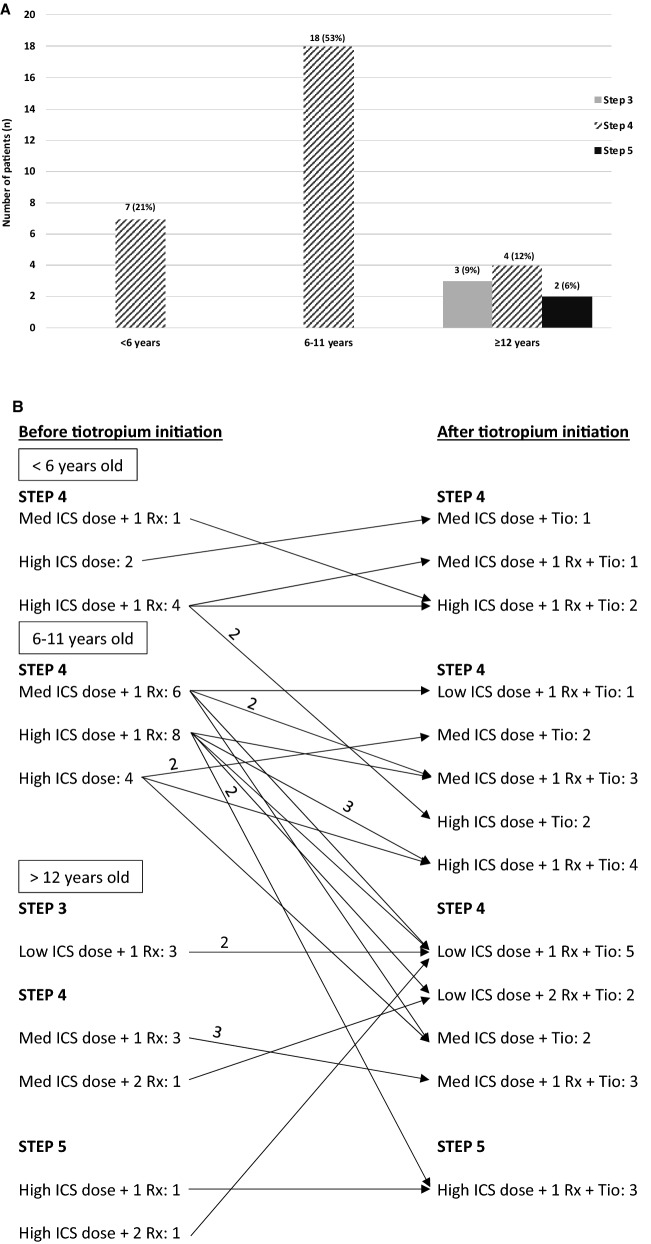
Treatment Steps prior and after the initiation of tiotropium. Panel A depicts the number (proportion) of children at each age-specific Step before, and Panel B, the change in therapy after, tiotropium (Tio) initiation, occurring in 1 or more than 1 (full arrow with the number of patients on top) patient. The number of adjunct therapy (Rx) added to a medium (MED) or high dose inhaled corticosteroids (ICS), is listed. Treatment steps were classified in accordance with the Global Initiative for Asthma (GINA); however, in children less than 6 years in whom there is no GINA Step 5, we assumed that those on Step 4, in whom tiotropium was an added adjunct therapy, have moved to Step 5

The 34 (11 females; 23 males) children had a median (range) age of 9.1 (1.4–17.8) years; 7 (21%) were less than 6 years, 18 (53%) between 6 and 11 years, and 9 (26%) were 12 years and older at tiotropium initiation. Spiriva Respimat® Softmist 2.5 ug 1 puff or 2 puffs once daily via a holding chamber or Spiriva Handihaler 18 ug 1 puff once daily were used in 1 (3%), 21 (62%), and 12 (35%) children, respectively. Using the GINA’s 2020 age-specific defined treatment steps [23] prior to tiotropium initiation, 3 (9%) children (which were all aged ≥ 12 years) were on GINA Step 3 therapy (low dose ICS with LABA, or leukotriene receptor antagonists (LTRA)), 29 (85%) on Step 4 with a medium ICS dose with LABA or LTRA or a high ICS dose and 2 (6%) on Step 5 with high dose ICS with LABA and/or LTRA (Fig. 1A and B). The initiation of tiotropium resulted in only 4 (13%) patients moving to a higher treatment GINA Step; 26 (84%) children maintained their initial treatment step either by replacing another adjunct therapy (LABA or LTRA) or by decreasing the ICS dose, and the remaining 1 (3%) child decreased his treatment step, by lowering daily ICS dose (Fig. 1B). In three patients, the treatment step after tiotropium initiation could not be determined because of missing information, namely one aged < 6 years old at step 4 (high ICS dose) therapy, one aged 6–11 years at step 4 (medium ICS dose + 1 adjunct therapy), and one aged > 12 years old at step 3 (low ICS dose + 1 adjunct therapy) before tiotropium initiation. Of note, although there is no GINA Step 5 for preschoolers, we assumed that all those on Step 4 in whom tiotropium was an added adjunct therapy moved to Step 5.

**Fig. 2:**
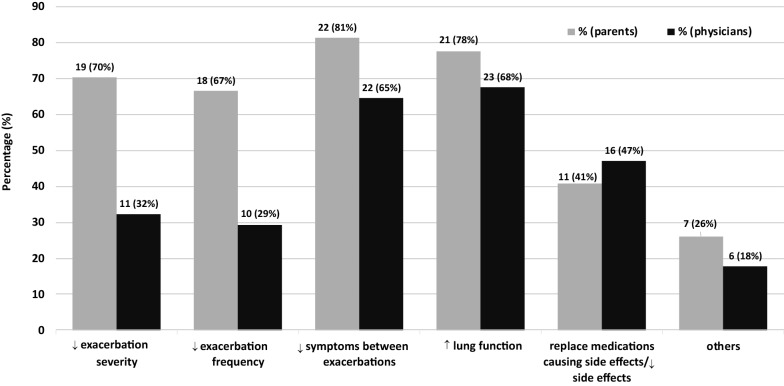
Treatment goals of the addition of tiotropium according to parents/children and physicians. This figure depicts the number (proportion) of children for each non-mutually exclusive indication as reported by parents in light, and by physicians in dark, bars. The denominator for each percentage was the number of children with completed questionnaires (27 parent/child and 34 physicians). In 96 and 74% of children, more than one indication was reported by parents and physicians, respectively

Treatment goals for the addition of tiotropium according to the parents/children and the physicians are depicted in Fig. 2. Physicians prescribed tiotropium, primarily to improve lung function in 23 (68%), improve control of interim (between-exacerbation) symptoms in 22 (65%) and/or reduce adverse effects on current therapy in 16 (47%), children. In addition to these, parents also primarily perceived tiotropium as a means to decrease the severity and frequency of exacerbations in 19 (70%) and 18 (67%) children, respectively. More than one indication was reported for 26/27 (96%) children by parents and 25/34 (74%) children by physicians. Of note, improving lung function or reducing adverse effects was the only physician-reported indication in 7 (21%) and 2 (6%) children, respectively. In fact, the only indication for initiating tiotropium in the 3 patients aged ≥ 12 years on GINA Step 3 before initiation, was to reverse an otherwise asymptomatic but seemingly none (or incompletely) reversible airway obstruction.

The main adverse effects on prior therapy motivating the initiation of tiotropium were; (i) growth retardation (9/16), attributed to fluticasone, fluticasone/salmeterol or mometasone furoate, (ii) adrenal insufficiency (1/16), attributed to fluticasone/salmeterol, or (iii) neuropsychiatric symptoms such as aggressivity (6/16) and sleep disorder (3/16), attributed to montelukast, fluticasone/salmeterol and/or budesonide/formoterol, and tachyphylaxis of long-acting beta-agonists (mentioned in the lessons learned by 2 physicians). In 91% (31/34) of children, tiotropium was prescribed as a means to replace or reduce the dose of the perceived offending molecule, most frequently the ICS (alone or in a fixed ICS/LABA combination), LABA (in a fixed combination) or LTRA. Other indications to add tiotropium included decreasing ICS/LABA dose to prevent adverse effects (4/34), preventing ICS dose escalation (1/34), and difficulty administering another drug (1/34). Physicians were comfortable prescribing tiotropium in 71% of patients, and most parents (89%; 24/27) were comfortable with initiating it.

**Fig. 3:**
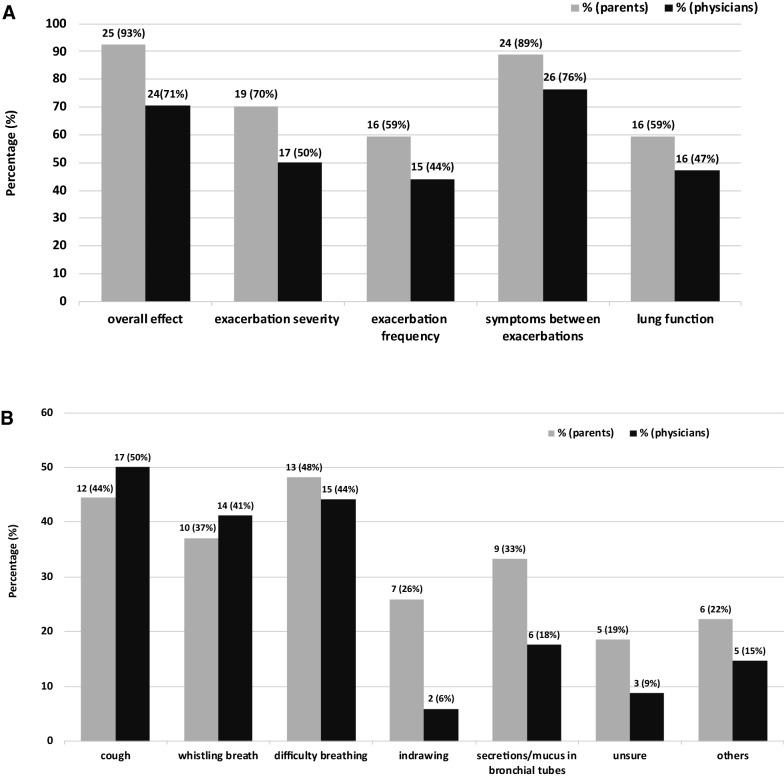
Improvements perceived by parents and physicians following the initiation of tiotropium. This figure depicts the number (proportion) of children for each non-mutually exclusive indicator of response in 3A and improved symptoms in 3B, as reported by parents in light bars and by physicians in dark bars. The denominator for each percentage was the number of children with completed questionnaires, namely 27 parent/child and 34 physician questionnaires

Overall, 93% of parents and 71% of physicians reported asthma improvement (+ 1 to + 3 on the 7-point Likert scale) after tiotropium initiation; no parent described deterioration (− 3 to − 1). Reported changes following initiation of tiotropium bromide were decreased interim symptoms in 76% and 89% children, according to physicians and parents, respectively, followed by decreased severity and frequency of exacerbations, and improved lung function (Fig. 3A). According to parents and physicians, the most improved symptoms were cough, breathing difficulty, whistling breath, and bronchial secretions/mucus. (Fig. 3B) Other perceived improvements included better control during exercise and reduction in thoracic pain.

Only 8/34 (24%) patients had stopped the medication prior to completing the survey. According to physicians, the main reasons for cessation were significant asthma improvement (3 [38%]), lack of improvement (2 [25%]), slight asthma deterioration (2 [25%]) and poor medication adherence (1 [13%]). After a trial of cessation, 5 (63%) children restarted tiotropium, following which improvement was reported in 3/5, no significant change in 1/5, and unsure in the remaining child.

No patient stopped using tiotropium because of adverse effects. Adverse effects were reported by parents in 2 (7%) children, one with xerostomia and one with insomnia. Physicians reported adverse effects in 2 (6%) children, one with constipation and one with insomnia; headaches were reported as an uncertain side effect in a third one. The overwhelming majority (93%) of parents and all physicians indicated that they would try tiotropium again based on their experience.

While most (93%) parents and physicians (82%) reported that the initiation of tiotropium to the treatment plan did not affect the parents’ ability to give other prescribed medications, some physicians raised caution about initiating another inhaler in patients with doubtful medication adherence and those taking numerous (anti-asthmatic or other) drugs.

Physicians were asked for the main lessons learned from initiating tiotropium. A majority mentioned the good improvement in chronic symptoms, particularly in children with prominent moist cough and bronchial secretions, followed by the ability to decrease the dose of ICS and/or LABA combination to lessen associated adverse effects (e.g., growth retardation, adrenal insufficiency, neuropsychiatric symptoms, tachyphylaxis etc.). Several physicians noted a significant improvement in lung function, indicating that they would try tiotropium again in the future, in children with none (or incompletely) reversible airflow obstruction despite optimized standard therapy. After gaining experience with several individual patient trials, most physicians mentioned increasing comfort with prescribing this drug, even if it remains off labeled for specific age groups or indications in Canada.

## Discussion

In this group of children, the physicians’ indications for prescribing tiotropium bromide were primarily to improve asthma control (interim symptoms), improve lung function, or alleviate perceived adverse events of the current treatment regimen. Parents’ main indication for initiation also included frequency and severity of exacerbations. Children were primarily at GINA step 4 or 5 prior to, and a majority (84%) maintained their treatment step after, tiotropium initiation; indeed, the latter most frequently replaced another adjunct or permitted a decrease in ICS dose. None of the patients’ asthma deteriorated because of tiotropium initiation.

Most parents and physicians perceived the initiation of tiotropium as effective and safe. It appeared particularly effective in decreasing the severity and frequency of asthma exacerbations as well as diminishing chronic symptoms, particularly moist cough, and bronchial secretions. These observations, predominantly documented in preschoolers and children under 12 years, are in line with a systematic review of 1902 children with asthma aged 1 to 17 years with moderate to severe symptomatic asthma, of which nearly half were aged 6 to 11 years, also attesting to significantly reduced chronic symptoms, improved lung function, and decreased exacerbation frequency [8]. More than 90% of parents/patients and all physicians mentioned they would try tiotropium again.

As per GINA recommendations, almost all patients in our study were at Step 4 or 5 [23] before tiotropium initiation; yet, few children increased their treatment step after its initiation. Indeed, tiotropium was used in about half of children as an alternative long-acting bronchodilator to LABA or alternative adjunct therapy to ICS because of adverse effects with current medications, such as neuropsychiatric effects attributed to montelukast [20], tachyphylaxis with LABA, or growth failure with a fixed ICS/LABA combination inhaler [24]. Poor tolerance or suboptimal efficacy of recommended standard therapy appeared as major motivators for clinicians to deviate from current pediatric guidelines by using tiotropium that remains off-label in Canada, and without stepping-up therapy, suggesting a specific niche of this drug, in light of its recognized safety profile [3].

Another possible indication appears to be patients presenting with seemingly none (or incompletely) reversible airway obstruction in patients on Steps 4 and 5 therapies, in whom improvement was observed. Recent data revealed promising perspectives for tiotropium as both an anti-inflammatory and anti-remodeling molecule, acting independently from the T2 inflammatory cascade, that could explain this improvement [25, 26]. Finally, patients with moist cough and abundant bronchial secretions between and during exacerbations appear to respond particularly well to tiotropium’s anticholinergic properties, suggesting another indication.

Tiotropium was very well tolerated with only a few displaying expected anticholinergic adverse effects, such as xerostomia, constipation, insomnia, and possibly headaches, all of which were only reported once. Those adverse effects appeared benign and self-limited and did not result in treatment cessation. These results are in line with data from a recently published review on tiotropium’s safety and tolerability, displaying an excellent safety profile in more than 6000 patients, with comparable proportions of patients reporting adverse events to those treated with placebo [4]. In children aged 1 to 5 years, the only published RCT testing the initiation of tiotropium versus placebo as an add-on to ICS as Step 3 option also reported a similar safety profile [7]. Given its reported safety profile, tiotropium probably deserves rigorous testing as a potential adjunct to ICS as Step 3 alternative therapy, compared to LABA or LTRA, neither of which has been formally tested in preschoolers.

This study’s strengths include the independent patients’ and physicians’ feedbacks based on real-life clinical data on the indication, efficacy, and safety in addition to information documented in medical charts. Most patients continued treatment beyond a year, allowing adequate time to evaluate key efficacy endpoints, namely exacerbations and chronic symptoms. We recognize several limitations. The small sample size without a control group as well as the absence of formal assessment of asthma control, treatment adherence and lung function in parental and physician questionnaires, prior and after treatment initiation, precludes firm conclusions on safety and efficacy. The retrospective design of the survey may have decreased precision and/or introduced a recall bias for both families and physicians, most of whom answered more than one year after initiation. Yet, more than two-thirds of responders indicated that the delay was unlikely to affect their response as three-quarters of children were still taking tiotropium at the time of the survey and all physicians had accessed the patient’s medical records to review indication and response. Of note, the identification of all eligible children based on a complete electronic database, with no patient omitted and an excellent participation rate, minimized the risk of selection bias. The online electronic questionnaires, conceived to alert responders of missing answers, resulted in complete questionnaires for all those initiated. An independent biostatistician analyzed the data. Although we cannot attest to accuracy when measuring parental perception and global assessment of response, based on objective and subjective indicators, the highly concordant response between parents’ and physicians’ perspectives collected independently suggests robustness in findings.

## Conclusions

In conclusion, these clinical observations suggest three additional pediatric indications, other than severe uncontrolled asthma, for considering the initiation of tiotropium, namely, to reverse seemingly none (or incompletely) reversible airway obstruction, alleviate perceived adverse events of the current treatment regimen while maintaining asthma control, and reducing moist cough and bronchial secretions. These observations are offered as hypothesis-generating that would need to be tested in prospective pediatric studies.

## Data Availability

The datasets generated and/or analyzed during the current study are available from the corresponding author of this study on reasonable request.

## Abbreviations

GINA: Global Initiative for Asthma
ICS: Inhaled corticosteroids
LABA: Long-acting beta_2_-agonist
LAMA: Long-acting muscarinic antagonist
LTRA: Leukotriene receptor antagonists
PFT: Pulmonary function test
RCT: Randomized control trial
